# Using Computational Methods to Improve Integrated Disease Management for Asthma and Chronic Obstructive Pulmonary Disease: Protocol for a Secondary Analysis

**DOI:** 10.2196/27065

**Published:** 2021-05-18

**Authors:** Gang Luo, Bryan L Stone, Xiaoming Sheng, Shan He, Corinna Koebnick, Flory L Nkoy

**Affiliations:** 1 Department of Biomedical Informatics and Medical Education University of Washington Seattle, WA United States; 2 Department of Pediatrics University of Utah Salt Lake City, UT United States; 3 College of Nursing University of Utah Salt Lake City, UT United States; 4 Care Transformation and Information Systems Intermountain Healthcare West Valley City, UT United States; 5 Department of Research & Evaluation Kaiser Permanente Southern California Pasadena, CA United States

**Keywords:** asthma, chronic obstructive pulmonary disease, decision support techniques, forecasting, machine learning

## Abstract

**Background:**

Asthma and chronic obstructive pulmonary disease (COPD) impose a heavy burden on health care. Approximately one-fourth of patients with asthma and patients with COPD are prone to exacerbations, which can be greatly reduced by preventive care via integrated disease management that has a limited service capacity. To do this well, a predictive model for proneness to exacerbation is required, but no such model exists. It would be suboptimal to build such models using the current model building approach for asthma and COPD, which has 2 gaps due to rarely factoring in temporal features showing early health changes and general directions. First, existing models for other asthma and COPD outcomes rarely use more advanced temporal features, such as the slope of the number of days to albuterol refill, and are inaccurate. Second, existing models seldom show the reason a patient is deemed high risk and the potential interventions to reduce the risk, making already occupied clinicians expend more time on chart review and overlook suitable interventions. Regular automatic explanation methods cannot deal with temporal data and address this issue well.

**Objective:**

To enable more patients with asthma and patients with COPD to obtain suitable and timely care to avoid exacerbations, we aim to implement comprehensible computational methods to accurately predict proneness to exacerbation and recommend customized interventions.

**Methods:**

We will use temporal features to accurately predict proneness to exacerbation, automatically find modifiable temporal risk factors for every high-risk patient, and assess the impact of actionable warnings on clinicians’ decisions to use integrated disease management to prevent proneness to exacerbation.

**Results:**

We have obtained most of the clinical and administrative data of patients with asthma from 3 prominent American health care systems. We are retrieving other clinical and administrative data, mostly of patients with COPD, needed for the study. We intend to complete the study in 6 years.

**Conclusions:**

Our results will help make asthma and COPD care more proactive, effective, and efficient, improving outcomes and saving resources.

**International Registered Report Identifier (IRRID):**

PRR1-10.2196/27065

## Introduction

### The Gap in Identifying Patients With Exacerbation-Prone Asthma and Patients With Exacerbation-Prone Chronic Obstructive Pulmonary Disease for Preventive Care

#### Management of Asthma and Chronic Obstructive Pulmonary Disease

**Figure 1:**
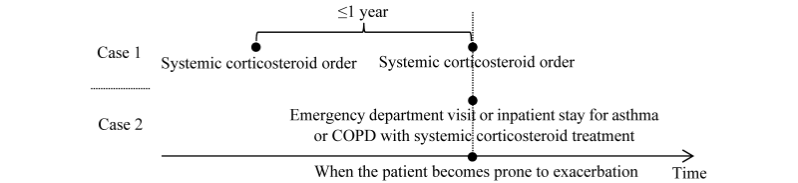
Determining when a patient with asthma or chronic obstructive pulmonary disease becomes prone to exacerbation. COPD: chronic obstructive pulmonary disease.

In the United States, 9.6% of children and 8% of adults have asthma, leading to 1.8 million emergency department visits, 493,000 inpatient stays, US $56 billion in cost, and 3630 deaths every year [1-4]. Approximately 6.5% of adults have chronic obstructive pulmonary disease (COPD), the third leading cause of death, leading to 1.5 million emergency department visits, 0.7 million inpatient stays, and US $32 billion in cost every year [5]. One main goal in managing patients with asthma and patients with COPD is to reduce exacerbations, which expend approximately 40% to 75% of their total care cost [6-8] and accelerate their lung function decline [9]. Approximately one-fourth of patients with asthma and patients with COPD are prone to exacerbation [10-14], meaning that a patient has (1) ≥2 systemic corticosteroid orders in a year or (2) ≥1 emergency department visit or inpatient stay for asthma or COPD with systemic corticosteroid treatment in a year (Figure 1) [10,13,15]. These patients incur approximately two-thirds of all exacerbations [12,13,16] and experience a low quality of life; sleep disturbance; limitations of daily activities impacting independence, relationships, family life, socialization, and career; anxiety; distress; missed work with lost earnings; missed school; high care costs; high hospital use; intubation; and death [10,17-19]. Even a brief use of systemic corticosteroids to treat exacerbations can greatly increase the risk of venous thromboembolism, sepsis, and fracture [20,21].

Many health care systems and health plans use predictive models as the best method [22] to identify high-risk patients for preventive care to improve outcomes and save resources [23-25]. For instance, this is the case with health plans in 9 of the 12 American metropolitan communities mentioned in the study by Mays et al [26]. However, no model exists to predict proneness to exacerbation, which only partly correlates with disease severity [16]. Exacerbation-prone patients are currently identified after exacerbations occur, making it too late to apply integrated disease management (IDM) for preventing exacerbations. IDM is defined as “a group of coherent interventions, designed to prevent or manage 1 or more chronic conditions using a community wide, systematic and structured multidisciplinary approach potentially employing multiple treatment modalities” [27]. IDM typically has several components, such as self-management education, skills training, care management, and structured follow-up [28,29]. Having a limited service capacity [29-33], IDM can lower hospital use by up to 40%; cut costs by up to 31%; greatly reduce symptoms; and enhance treatment adherence, patient satisfaction, and quality of life by 30%-60% [26,28-32,34-42]. Neither patient registries nor dashboards are able to identify exacerbation-prone patients before exacerbations occur and, thus, to apply IDM in a timely manner. A patient registry tracks a given patient cohort but cannot make predictions. Although many attributes are often needed to achieve high prediction accuracy [43-45], a dashboard tracks only a few attributes. To have prediction capability, a dashboard needs to be supported by a predictive model in the backend. Models for proneness to exacerbation are needed to guide the use of IDM and to prevent exacerbations. This cannot be done well with the current model building approach for other asthma and COPD outcomes, which has 2 major gaps due to the limited use of temporal features showing early health changes and general directions [46-94]. Each temporal feature is an independent variable computed on one or more longitudinal attributes, such as the slope of pulmonary function last year, the slope of BMI last year, the number of days in the previous week during which the sulfur dioxide level was ≥4 parts per million, and whether the patient’s filling frequency of oral corticosteroid prescription increased over time. Although this study focuses on exacerbation-prone asthma and COPD as use cases, the proposed computing techniques and software can be harnessed to forecast outcomes of other diseases such as congestive heart failure and diabetes, with temporal features such as the slopes of cardiac function and blood glucose level over time.

#### Gap 1: Low Prediction Accuracy

Existing models for predicting an individual asthma or COPD patient’s health outcomes typically have low accuracy [46-94]. The systematic review by Loymans et al [52] and our review [43] showed that for forecasting hospital use (emergency department visits and inpatient stays) for asthma in patients with asthma, each previous model, excluding the models of Zein et al [58], has an area under the receiver operating characteristic curve (AUC) within 0.61-0.81, a sensitivity within 25%-49%, and a positive predictive value within 4%-22% [46-57]. The models of Zein et al [58] and our recent new models [43-45] have similarly higher accuracy but are still not good enough for aligning preventive care with the patients needing it the most. The case with COPD is similar [59-94].

Existing models for predicting asthma and COPD outcomes typically have low accuracy for several reasons:

1. Existing models use elementary temporal features such as the count of inpatient stays and ever intubated last year, but they rarely use more advanced temporal features such as the slope of the number of days to albuterol refill showing general directions. Many highly predictive temporal features are yet to be identified or are unused. In 2018, Google used all of the attributes in the electronic medical record along with long short-term memory (LSTM) [95,96], one type of deep neural network, to discover temporal features automatically from longitudinal data [97]. This raised the AUC by approximately +10% for projecting each of long hospital stay, in-hospital mortality, and unanticipated readmissions in 30 days [97]. Several other studies [98-100] obtained similar results for various clinical prediction tasks. This matches recent progress in areas such as video classification, speech recognition, and natural language processing, where temporal features LSTM automatically discovered from data beat those that experts provided or other temporal and sequential pattern mining methods [101-104] mined from data. The LSTM model of Xiang et al for predicting asthma outcome [57] had a low AUC of 0.7 because it used only 3 types of attributes and mostly inpatient data without much outpatient data, not because LSTM is ineffective.
2. Although >100 potential risk factors for poor outcomes in asthma and COPD are known [50-52,105-112], a typical previous model uses only a few (eg, ≤17) [46-57,59-93]. None of the published models adopt all established risk factors contained in contemporary electronic medical records [113].
3. Weather and air quality variables impact asthma and COPD outcomes [114-117], but they are seldom used in existing models.

#### Gap 2: No Information Given on the Reason Why a Patient is Deemed High Risk and the Potential Interventions to Reduce the Risk

To provide preventive care well, clinicians need to know the reason a patient is deemed high risk and the potential interventions to reduce the risk. Sophisticated predictive models, including the bulk of machine learning models such as LSTM, are black boxes and provide no such information, although explanation is critical for users’ acceptance, satisfaction, trust, and decision correctness [118-121]. Often, a patient’s clinical records include numerous variables on many pages recorded over multiple years [122]. As the model gives no explanation, already occupied clinicians need to expend extra time on chart review to identify the reasons. This is difficult and time consuming. In fact, the black-box issue has been a major reason for the slow adoption of machine learning in clinical practice, despite machine learning often producing the highest prediction accuracy among all predictive modeling methods [33,123-127].

A clinician can develop a care plan using subjective, variable clinical judgment. However, this care plan often misses some suitable interventions because of the following reasons:

1. Big practice variation, frequently by 1.6-5.6 times, shows up across facilities, clinicians, and regions [128-135].
2. A patient can become high risk for many reasons, each shown by a risk pattern given by a feature combination, for example, the sulfur dioxide level was ≥4 parts per million for ≥4 days in the previous week and the number of days to albuterol refill rose over 12 months. Many features and feature combinations exist. A clinician is a human, can typically process ≤9 information items at once [136], and can easily miss some key reasons for which the patient is high risk. Outcomes can degrade if suitable interventions are not used. Regular automatic explanation methods [137-140] cannot deal with longitudinal data and address this issue well.

### Our Proposed Solutions

To enable more patients with asthma and patients with COPD to obtain suitable and timely care to prevent exacerbations, we will (1) use temporal features to develop the first set of models to accurately predict exacerbation-prone asthma and COPD, (2) automate finding modifiable temporal risk factors for every high-risk patient, and (3) assess the impact of actionable warnings on clinicians’ decisions to use IDM to prevent proneness to exacerbation.

### Innovation

We will develop new techniques to automate the extraction of temporal features from longitudinal data and explain machine learning predictions on longitudinal data. We will improve preventive care, notably for asthma and COPD, by steering it to the patients who need it more precisely and in a more timely manner than the current risk modeling methods:

1. To the best of our knowledge, this study will construct the first set of models to predict which patients with asthma and which patients with COPD will be prone to exacerbation. Currently, these patients are found after exacerbations occur, making it too late to apply IDM for preventing exacerbations. This is a major public health issue [29,31,32]. Our models can improve IDM and guide its use to avert exacerbations. Compared with the current model building method for other asthma and COPD outcomes that often produces low accuracy, our model building method will lead to more accurate predictions.
2. To the best of our knowledge, this will be the first study to extract comprehensible and predictive temporal features semiautomatically from longitudinal data without needing any manually prespecified pattern template, which is required by many sequential and temporal pattern mining methods [102-104]. This helps raise the model accuracy and reduce the effort required to construct clinically usable models. At present, clinicians usually have to manually identify such features to construct such models. However, this is time consuming and difficult. Previous models for asthma and COPD rarely use more advanced temporal features, such as slope [46-94]. In addition, although current deep neural network methods can automatically discover temporal features, the discovered features are hidden in neurons and are often incomprehensible, making it difficult to explain the predictions [137,138].
3. To the best of our knowledge, this will be the first study to automate giving rule-formed explanations for machine learning predictions directly on longitudinal data. Clinicians need explanations to understand the predictions and decide IDM enrollment and interventions. Rule-formed explanations are easier to comprehend and can better hint at actionable interventions than other forms of automatic explanations. Most automatic explanation methods [137,138] for machine learning predictions cannot deal with longitudinal data. Our previous automatic explanation method [140-142] is no exception. It has 5 hyperparameters whose effective values vary by modeling problem and data set. A computing expert often requires several months to perform many trials to find these values laboriously for a data set. We will improve our previous method to deal with longitudinal data and automatically and efficiently select hyperparameter values; therefore, health care researchers with limited computing expertise can use our method with low overhead.
4. To the best of our knowledge, this will be the first study to automate finding modifiable temporal risk factors and recommending interventions on the basis of objective data, making IDM more efficient and effective. At present, clinicians rely on subjective, variable judgment to create care plans manually and overlook some suitable interventions for high-risk patients.
5. To the best of our knowledge, this will be the first study to assess the impact of actionable warnings on clinicians’ decisions to use IDM to prevent proneness to exacerbation.

## Methods

### Computing Resources

We will conduct all experiments on a password-protected and encrypted computer cluster hosted at the University of Washington Medicine (UWM). With appropriate authorization and using their university computers, all research team members and test participants at UWM can remotely access this computer cluster.

### Data Sets

All data that will be used in this study are structured. We will use clinical and administrative data stored in the enterprise data warehouses of 3 prominent American health care systems: UWM, Kaiser Permanente Southern California (KPSC), and Intermountain Healthcare (IH). We will use >200 clinical and administrative variables listed in our papers’ [43-45] appendices, with differing names of the same concept in distinct electronic medical record systems already manually matched by us. These variables cover a wide range of aspects, such as patient demographics, encounters, medications, laboratory tests, diagnoses, procedures, vital signs, and allergies. We can form the temporal features of most variables, which are longitudinal with timestamps.

In Utah, IH is the largest health care system, with 24 hospitals and 215 clinics. As in our previous work on asthma outcome prediction [43-45], an IH data analyst will run Oracle database queries to retrieve a deidentified IH data set (eg, shift dates, replace identifiers, and replace ages that are ≥90 years) and use Secure Shell (SSH) to encrypt it and transfer it to the password-protected and encrypted computer cluster, where we will perform analysis. The IH data set covers patient encounters from 2005 to 2020. For the previous 5 years, data for children cover >5000 pediatric patients with asthma (aged <18 years) per year. Data for adults cover >14,000 adult patients with asthma (aged ≥18 years) and >6000 adult patients with COPD per year. IH expends many resources on data integrity and accuracy. Owing to its large size and variable richness [143], the data set offers many advantages for exploring the proposed methods.

UWM and KPSC have similar strengths. In Washington, UWM is the largest academic health care system, with 4 hospitals and 12 clinics for adults. A UWM data analyst will execute SQL Server database queries to retrieve a deidentified UWM data set (eg, shift dates, replace identifiers, and replace ages that are ≥90 years) and use SSH to encrypt it and transfer it to the password-protected and encrypted computer cluster. The UWM data set covers adult patient encounters from 2011 to 2020. For the previous 5 years, data cover >12,000 adult patients with asthma and >5000 adult patients with COPD per year.

In Southern California, KPSC is the largest integrated health care system, with 15 hospitals and 231 clinics [144]. A KPSC data analyst will run database queries to retrieve a deidentified KPSC data set (eg, shift dates, replace identifiers, and replace ages that are ≥90 years) and use SSH to encrypt it and transfer it to the password-protected and encrypted computer cluster. The KPSC data set covers patient encounters from 2009 to 2020. For the previous 5 years, data for children cover >77,000 pediatric patients with asthma per year. Data for adults cover >172,000 adult patients with asthma and >78,000 adult patients with COPD per year.

In addition to the clinical and administrative data, we will adopt 11 weather and air quality variables that we have downloaded from public sources [145,146]: daily mean particulate matter ≤2.5 μm in diameter, daily maximum 8-hour carbon monoxide, daily mean particulate matter ≤10 μm in diameter, daily maximum 8-hour ozone, daily maximum 1-hour nitrogen dioxide, daily maximum 1-hour sulfur dioxide, hourly mean precipitation, hourly mean relative humidity, hourly mean wind speed, hourly mean temperature, and hourly mean dew point. These variables were recorded over 16 years (2005-2020) by monitoring stations located in the areas covered by IH, UWM, and KPSC.

The following discussion focuses on asthma. Whenever we refer to asthma, the same applies to COPD.

### Aim 1: Use Temporal Features to Accurately Predict Exacerbation-Prone Asthma and COPD

We will extract comprehensible and predictive temporal features semiautomatically from patient, weather, and air quality data and construct models to predict proneness to exacerbation. Each feature uses ≥1 raw variable. There is an almost infinite number of possible features. Traits of pediatric patients’ parents and other factors could also impact patient outcomes. Our goal is not to test all possible useful features and obtain the theoretically maximum possible prediction accuracy. Instead, we intend to show that temporal features can be used to improve prediction accuracy and IDM. We will create a separate model for every disease and health care system pair. This study will focus on associations, as is sufficient for decision support for IDM and common with predictive modeling.

#### Data Preprocessing

All data sets will be converted into the Observational Medical Outcomes Partnership (OMOP) common data model format [147] and its linked standardized terminologies [148]. Much of the UWM data are already in this format. IH and KPSC have provided their data in an internal normalized format that is similar to this format. We will expand the data model to include patient, weather, and air quality variables that the original data model misses but exist in our data sets. We will use the method described in our paper [149] to choose the most pertinent laboratory tests. To reduce the number of features, we will use the Agency for Healthcare Research and Quality Clinical Classifications Software system [150,151] to merge diseases, use the Berenson-Eggers Type of Service system [152] to merge procedures, and use the Hierarchical Ingredient Code 3 system [153] to merge drugs. We will adopt the method used in our previous work [43-45] to identify, correct, or delete invalid values. To deal with missing values, we will test various imputation techniques [154,155], such as the last observation carried forward, replacement with mean values, and replacement with median values, and use the technique that works the best.

The patient, weather, and air quality variables will be used. The patient variables will cover standard variables studied in the clinical predictive modeling literature [128,129,154], such as diagnoses, and >100 known potential risk factors for poor asthma outcomes listed in our papers [43-45,156]. One such risk factor is the frequency of nighttime awakening recorded on the validated Asthma Control Test questionnaire [157] in the electronic medical record system. For weather and air quality variables, we will perform inverse distance weighting spatial interpolation [158] to compute their daily average values at the patient’s residence zip code from their values at local monitoring stations, as we and others did before for asthma outcome prediction [159-161].

#### Asthma and COPD Cases and Outcomes

We will implement and test our method using (1) pediatric asthma, (2) adult asthma, and (3) COPD. We will use our previous method [44] adapted from the literature [47,162,163] to identify patients with asthma. We deem a patient to have asthma in a given year if the patient has ≥1 asthma diagnosis code (*International Classification of Diseases, Ninth Revision* [*ICD-9*] 493.x or *International Classification of Diseases, Tenth Revision* [*ICD-10*] J45 and J46.x) in the year. The outcome is whether the patient became prone to exacerbation (ie, had either ≥2 systemic corticosteroid orders or ≥1 emergency department visit or inpatient stay with a principal diagnosis of asthma and systemic corticosteroid treatment) in the following year [10,15].

**Figure 2:**
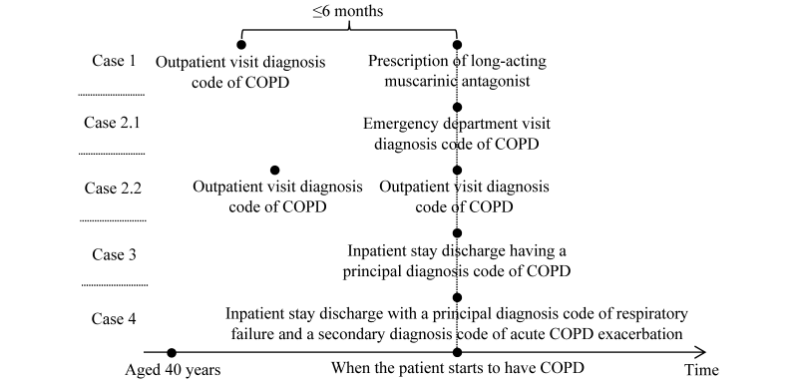
Determining when a patient starts to have chronic obstructive pulmonary disease. COPD: chronic obstructive pulmonary disease.

We will use our previous method [164] adapted from the literature [165-168] to identify patients with COPD. As shown in Figure 2, we deem a patient to have COPD if the patient is aged ≥40 years and fulfills any of the following 4 conditions:

1. An outpatient visit diagnosis code of COPD (*ICD-9*: 491.22, 491.21, 491.9, 491.8, 493.2x, 492.8, and 496; *ICD-10*: J42, J41.8, J44.x, and J43.x), followed by ≥1 prescription of long-acting muscarinic antagonists (aclidinium, glycopyrrolate, tiotropium, and umeclidinium) within 6 months
2. ≥1 emergency department or ≥2 outpatient visit diagnosis codes of COPD (*ICD-9*: 491.22, 491.21, 491.9, 491.8, 493.2x, 492.8, and 496; *ICD-10*: J42, J41.8, J44.x, and J43.x)
3. ≥1 inpatient stay discharge with a principal diagnosis code of COPD (*ICD-9*: 491.22, 491.21, 491.9, 491.8, 493.2x, 492.8, and 496; *ICD-10*: J42, J41.8, J44.x, and J43.x)
4. ≥1 inpatient stay discharge with a principal diagnosis code of respiratory failure (*ICD-9*: 518.82, 518.81, 799.1, and 518.84; *ICD-10*: J96.0x, J80, J96.9x, J96.2x, and R09.2) and a secondary diagnosis code of acute COPD exacerbation (*ICD-9*: 491.22, 491.21, 493.22, and 493.21; *ICD-10*: J44.1 and J44.0) [164].

The outcome is whether the patient became prone to exacerbation (ie, had either ≥2 systemic corticosteroid orders or ≥1 emergency department visit or inpatient stay with a principal diagnosis of COPD and systemic corticosteroid treatment) in the following year [13].

#### Extracting Temporal Features

We will adopt the method described in our design paper [149] to extract comprehensible and predictive temporal features semiautomatically from longitudinal data. In aim 1, we will use the extracted features to construct the final predictive models. In aim 2, we will use the extracted features to automate finding modifiable temporal risk factors for every high-risk patient. The main idea of our temporal feature extraction method is to build a so-called multi-component LSTM deep neural network model on longitudinal data, use a so-called exclusive group Lasso (least absolute shrinkage and selection operator) regularization method to restrict the number of attributes used in each component LSTM network, and then perform visualization to identify comprehensible temporal features from certain cell vector elements in each component LSTM network. The final step of using visualization to identify temporal features and providing their definitions involves humans and is semiautomatic. All other steps are automatic. Our temporal feature extraction method is general and can be used for many clinical applications. Our method has never been implemented in computer code. In addition, some of its technical details are not provided in our design paper [149]. In this study, we will fill in all of the missing technical details and code and test this method.

#### The Final Predictive Models in Aim 1

We will use the extracted temporal features, such as the slope of the number of days to albuterol refill, to transform longitudinal data into tabular data, producing 1 column per temporal feature, and add static features. We will place no artificial upper or lower bound and use as many features as needed (likely several dozen to several hundred features based on our previous experience [43-45]). Our data are relatively balanced [10-14]. We will harness Weka [169], a major open-source machine learning toolkit, to create the final models in aim 1. As aim 2 shows, these models are suitable for automatic explanations. Weka implements many classic machine learning algorithms and feature selection techniques. We will adopt supervised algorithms and our previous method [170] to automate selection of the machine learning algorithm, feature selection technique, and hyperparameter values out of all applicable ones. When needed, we will manually perform fine-tuning.

We will use past data up to the prediction time point to construct 5 sets of models, 1 set for each of 5 combinations: pediatric asthma at IH and KPSC and adult asthma at IH, UWM, and KPSC. UWM has rather incomplete data on many of its patients, partly because most of its patients are referred from elsewhere. To reduce the impact of incomplete data on model performance, we will harness our previous constraint-based method [164,171] to identify the patients apt to get most of their care from UWM, and we will construct models for them. As mentioned earlier, we will also implement and test our method on COPD.

#### Evaluating Model Performance and Power Analysis

The discussion below focuses on IH data. The cases with UWM and KPSC data are analogous. As we need to calculate outcomes in the following year, we effectively have 15 years of IH data over the previous 16 years. We will train and test the models in a standard way. On the data of the first 14 years, we will perform stratified 10-fold cross validation [169] to train models and gauge their performance. On the data of the 15th year, we will appraise the performance of the best models, reflecting future use in practice. We will use the standard performance metric AUC [169] to choose the best model and record its AUC. We will show the model’s accuracy, sensitivity, specificity, and positive and negative predictive values when the cutoff threshold of binary classification varies from the top 1% to the top 50% of patients with asthma with the highest predicted risk. To find the variables essential for achieving high model performance, backward elimination [154] will be adopted to remove features as long as AUC drops by ≤0.002. We will compare the variables essential for achieving high model performance on IH data with those on UWM and KPSC data. The gender’s predictive power will be checked explicitly. We will use the variables appearing in both the UWM and IH data sets to construct a best model on IH data and compare its performance on UWM data with that on IH data. We will use the variables appearing in both the KPSC and IH data sets to construct a best model on IH data and compare its performance on KPSC data with that on IH data.

We will test the hypothesis that adopting our techniques could enhance model performance twice, once for adults and once for children. To do this, we will compare the AUCs of 2 predictive models built using the attributes in our data set and the best machine learning algorithm. The first model will harness all the features essential for achieving high model performance. The second model will be performed in the same way as our recent model for predicting hospital use for asthma [44] related to proneness to exacerbation. We anticipate that the second model will have an AUC around our recent model’s AUC of 0.86. Our hypothesis is as follows:

1. Null hypothesis: the second model has the same AUC as the first model.
2. Alternative hypothesis: the second model has a smaller AUC than the first model.

The categorical outcome variable of proneness to exacerbation has 2 values (classes). According to the standard method developed by Obuchowski and McClish [172] for AUC-related sample size computation, using a 2-sided Z test at a significance level of 0.05 and assuming for both classes a Pearson correlation coefficient of 0.6 between the 2 models’ predictions, a sample size of 464 instances per class provides 90% power to identify an AUC difference of 0.05 between the 2 models. The 15th year’s IH data cover >5000 children with asthma and >14,000 adults with asthma, offering sufficient power to test our hypothesis. If the real correlation coefficient is different from the assumed one by no more than a moderate degree, the conclusion would remain valid.

#### Sensitivity Analysis

IH, UWM, and KPSC each recorded many variables. Another health care system could record fewer variables. We will test miscellaneous variable combinations and assess the performance of the corresponding modified models. This will help us ensure generalizability and identify critical variables. If a health care system does not record a particular critical variable, the assessed performance numbers can suggest alternative variables with minimal degradation of model performance. On the basis of our clinical experts’ judgment, we will merge variables apt to co-occur, such as the variables appearing in a lab test panel, into groups. We will form and publish a table listing possible combinations of variables by groups, accompanied by the performance numbers and the trained parameters of the corresponding predictive models. A health care system interested in deploying the model can use the table to assess the expected model performance for their data environment and determine the variables to be recorded. The table contains a distinct column for each of IH, UWM, and KPSC. Many variables recorded by IH, UWM, and KPSC and used in this study are common and recorded by many other health care systems. Hence, these health care systems already have all the variables appearing in each of many rows in the table.

### Aim 2: Automate Finding Modifiable Temporal Risk Factors for Every High-Risk Patient

#### Overview of Aim 2

For patients with predicted risk over a fixed bar, such as the 75th percentile, we will automate explaining warnings, finding modifiable temporal risk factors, and recommending customized interventions. This will help clinicians make decisions regarding IDM enrollment and develop customized care plans. To create the new function, we will enhance our previous method [140] of automatically explaining machine learning predictions with no loss of model performance. Our previous method cannot deal with longitudinal data, has hard-to-tune hyperparameters, and has not been previously used for COPD or IDM.

#### Explanation Method

As aim 1 shows, we will use temporal features to transform longitudinal data into tabular data, producing one column per temporal feature. Our previous automatic explanation method [140] can then be used. Each patient is labeled as either high risk or not high risk. Our method mines from past data association rules tied to high risk. One example rule is as follows: the sulfur dioxide level was ≥4 parts per million for ≥4 days in the previous week AND the number of days to albuterol refill rose over the previous 12 months → the patient is high risk. The second item on the left-hand side of the rule is a modifiable temporal risk factor. Three interventions for it are to (1) assess the patient on asthma triggers and ensure that the patient avoids them; (2) evaluate compliance with asthma controller medications and prescribe, modify, or increase the doses of the medications if necessary; and (3) create a new asthma action plan to use more aggressive interventions when the patient is in the yellow zone [173]. Our paper [149] presented multiple interventions for several other temporal risk factors. Through discussion and consensus, our clinical team will examine the mined rules and remove those that make little or no clinical sense. For each rule left, our clinical team will identify the modifiable temporal risk factors in the rule and provide zero or more evidence-based interventions from the literature addressing the reason that the rule provides. The rules are used to provide explanations instead of predictions.

At prediction time, for each patient our most accurate model (initially resulting from aim 1) marks high risk, we will identify and present all association rules tied to high risk and whose left-hand side conditions are fulfilled by the patient, as well as show the rules’ linked interventions as our recommendations. Every rule presents a reason why the patient is predicted to be at high risk. Users of the automatic explanation function could provide input to facilitate the identification and removal of unreasonable rules [174].

#### Automatically and Efficiently Selecting Hyperparameter Values

Our previous automatic explanation method [140-142] uses 5 hyperparameters. Their effective values differ according to the modeling problem and data set. In our previous work [140-142], for each data set, a computing expert took several months to perform many trials to laboriously find these values. To reduce this overhead and to allow health care researchers with no extensive computing background to use our method, we will extend the progressive sampling-based approach, which we previously developed for expediting automatic machine learning model selection [170], to automatically and efficiently select the values of the 5 hyperparameters. On average, our progressive sampling-based approach performs the search process 2 orders of magnitude faster than the modern Auto-Weka automatic selection approach [170,175]. Our approach generalizes to many clinical applications.

We will also develop our techniques on COPD.

### Aim 3: Assess the Impact of Actionable Warnings on Clinicians’ Decisions to Use IDM to Prevent Proneness to Exacerbation

#### Goal of Aim 3

To prepare for future clinical use, in a UWM test setting, we will assess the impact of actionable warnings on clinicians’ decisions to use IDM in patients with asthma to prevent proneness to exacerbation. We will also access UWM physicians’ (primary care doctors, pulmonologists, and allergists) and nurses’ subjective opinions of automatic explanations.

#### Recruiting Subjects

As an UWM operational project, we are building asthma outcome prediction models and have access to approximately 700 physicians and approximately 1700 nurses managing adult patients with asthma. Through personal contact and advertising in their email lists, we will recruit 20 test participants (10 physicians and 10 nurses) with purposeful sampling to guarantee sufficient variability in their work experience [176]. Every test participant will offer consent before participation and be current on UWM’s policy training on information security and privacy. To protect privacy, every test participant will receive a pseudonym linking their responses. Upon task completion, each physician will receive US $2300 as compensation for participation and for approximately 20 hours of work. Each nurse will receive US $1200 as compensation for participation and for approximately 20 hours of work.

#### Procedures

Using the 15th year’s (2019) IH data, we will randomly select 800 IH adult patients with asthma and automatically explain the predictions of the best performing IH model formed in aim 1. Using patients outside the UWM can help ensure that no test participant knows the outcome of any of these patients in the following year. We will present a distinct subset of 40 patients to each test participant and proceed in the following 4 steps:

1. Step 1: For each patient, we will display to the test participant the 2005-2019 deidentified patient data in reverse chronological order, as in the electronic medical records, and ask the test participant to write down the IDM enrollment decision (yes or no) and any interventions that the test participant plans to adopt on the patient.
2. Step 2: For each patient, we will display to the test participant the 2005-2019 deidentified patient data, the prediction, the automatic explanations, and the interventions connected to them. We will ask the test participant to write down their IDM enrollment decision (yes or no) on the patient after seeing the prediction and the explanations, the linked interventions they agree with, those they disagree with, and the interventions that they come up with in step 1 but whose concepts are missed by the linked interventions.
3. Step 3: Perceived usefulness is closely linked to future use intentions and actual function use [177,178]. Using the classic Technology Acceptance Model satisfaction questionnaire [179], we will survey the test participant to know their perceived ease of use and usefulness of automatic explanations.
4. Step 4: We will conduct a focus group with 10 randomly chosen test participants to assess what helps them use or prevents them from using the automatic explanations in clinical practice and why they agree or disagree with the automatically recommended interventions.

#### Quantitative and Qualitative Analyses

##### Quantitative Analyses

We will provide descriptive statistics for each quantitative outcome measure, including the mean and SD of each of the following: (1) the number of times that a test participant changes their IDM enrollment decision on a patient after seeing the prediction and the explanations, (2) the number of linked interventions for a patient a test participant agrees with, (3) the number of linked interventions for a patient a test participant disagrees with, (4) the number of interventions that a test participant comes up with for a patient in step 1 but whose concepts are missed by the linked interventions, and (5) the rating of every item in the Technology Acceptance Model satisfaction questionnaire. We will test the hypothesis that giving actionable warnings will improve clinicians’ decision to use IDM to prevent proneness to exacerbation, that is, the degree of IDM enrollment decision matching whether the patient will become prone to exacerbation in the following year. Our hypothesis is as follows:

1. Null hypothesis: The degree of IDM enrollment decision matching whether the patient will become prone to exacerbation in the following year in step 2 is the same as that in step 1.
2. Alternative hypothesis: The degree of IDM enrollment decision matching whether the patient will become prone to exacerbation in the following year in step 2 is larger than that in step 1.

We will fit a random effect logistic model that accounts for the correlation among the outcomes related to the same test participant.

##### Power Analysis for the Quantitative Analyses

Assuming a modest intraclass correlation of 0.1 within the same test participant on the outcome, a sample size of 40 patients per test participant for the 20 test participants is equivalent to a total of 82 independent patients after factoring in the clustering effect. We will have, at a 2-sided significance level of .05, 80% power to detect a 9.7% increase in the chances of improving clinicians’ decisions to use IDM with actionable warnings. If the real correlation is different from the assumed one by no more than a moderate degree, a similar conclusion would hold.

##### Qualitative Analyses

Using the inductive method described in Patton et al [176,180], test participants’ comments recorded in text during the focus group will be loaded into ATLAS.ti qualitative analysis software (ATLAS.ti Scientific Software Development GmbH) [181]. Three people from our research team will highlight the quotations independently. Through discussion and negotiated consensus in multiple iterations, these people will review quotations, categorize quotations into precodes, merge codes into categories, and synthesize categories to identify general themes.

#### Exploring for Other Diseases

Preventive care is also widely adopted for patients with heart diseases and diabetes. To explore what will be needed to generalize our techniques to predict outcomes of these diseases in the future, we will conduct 2 phases of focus groups, each phase with a distinct set of 6 UWM clinical experts on these diseases, and add more phases if these 2 phases do not reach saturation.

As stated immediately before aim 1, the discussion above concentrates on asthma. Whenever we refer to asthma, the same applies to COPD and will be implemented and tested on COPD in aims 1 and 2 but not in aim 3.

### Ethics Approval

We have received approval from the UWM institutional review board for this study and are applying for approval from IH and KPSC.

## Results

We have downloaded 2005-2020 weather and air quality data from public sources [145,146]. For the clinical and administrative data, GL at UWM has obtained the 2005-2018 data of patients with asthma from IH [44], the 2009-2018 data of patients with asthma from KPSC [45], and the 2011-2018 data of patients with asthma from UWM [43]. We are retrieving the other clinical and administrative data, mostly of patients with COPD, from IH, UWM, and KPSC. We intend to complete the study in 6 years.

## Discussion

### Using Our Results in Clinical Practice

IH, UWM, KPSC, and many other health care systems use IDM and use inaccurate predictive models with AUC<0.8 and sensitivity ≤49% for preventive care via care management [22,24-26,46-57,59-93]. Similar to our recent work of using IH, UWM, and KPSC data to greatly increase prediction accuracy for hospital use for asthma [43-45] related to exacerbation proneness, we expect our models predicting exacerbation proneness to be more accurate than those inaccurate models, benefit many patients, and have practical value. We will automate explaining warnings and recommending interventions to aid clinicians to review structured data in patient clinical records faster and create customized care plans based on objective data. After our methods find patients with the greatest predicted risks and offer explanations, clinicians will review patient clinical records, look at factors such as social dimensions [182], and make IDM enrollment and intervention decisions. As feature patterns linked to high risk and patient status keep changing, our techniques can be used continuously to move patients out of and into IDM and to discover new feature patterns.

In addition to making the predictive model more accurate, using temporal features showing early health changes and general directions could also boost warning timeliness. If a patient will be admitted to the hospital for COPD or asthma and the model would not predict this until 1 week before the hospital admission, intervening at that time could be too late to avoid the admission. If the model uses suitable temporal features and runs continuously, this patient could be found several weeks earlier, when health decline just begins and preventing hospital admission is likely.

### Generalizability

Predictive models vary by diseases and other factors and could be dissimilar to each other. However, our proposed methods and software for extracting temporal features and automatically explaining machine learning predictions are general and do not rely on any special property of a specific health care system, disease, or patient cohort. Given a new data set with a different disease, set of variables, patient cohort, or prediction target, one can plug in our software to extract temporal features and to automatically explain machine learning predictions. Besides being used for patients with asthma and patients with COPD, preventive care is also widely adopted for patients with heart disease and patients with diabetes [128], where our techniques could be harnessed, for example, to predict hospital use. Our sensitivity analysis results in aim 1 can be used to identify critical variables and determine how to generalize a predictive model to a health care system recording a different set of variables from IH, UWM, and KPSC.

We will use data retrieved from 3 health care systems, UWM, IH, and KPSC, to demonstrate our techniques on patients with asthma and patients with COPD. These systems include an academic system that has most of its patients referred from elsewhere (UWM), 2 integrated systems (IH and KPSC), and 42 hospitals and 458 clinics. Spreading across 3 large geographic areas, these heterogeneous facilities range from tertiary care hospitals in large cities served by subspecialists to community rural and urban clinics served by general practitioners and family physicians with limited resources. These health care systems use 4 distinct electronic medical record systems: KPSC uses Epic; UWM uses Epic and Cerner; and IH uses Health Evolution through Logical Processing, Health Evolution through Logical Processing 2, and Cerner. Variations in health care system type, patient population, geographic location, cultural background, staff composition, electronic medical record system, and scope of services enable us to identify factors that generalize to other facilities nationwide. The OMOP common data model [147] and its linked standardized terminologies [148] standardize administrative and clinical variables from ≥10 major American health care systems [183,184]. Our models will be based on OMOP and apply to these health care systems using OMOP.

With appropriate extension, our techniques can be adopted for miscellaneous diseases and decision support applications and can improve clinical machine learning. For example, our techniques can enhance the prediction accuracy of other outcomes such as no-shows [185], hospital use [186], and treatment adherence [187]. This will enable us to target resources, such as telephone reminders to reduce no-shows [185], home visits by nurses and care management to reduce hospital use [186], and interventions to boost treatment adherence [187].

We can use the features extracted by our temporal feature extraction method to create a feature library to ease feature reuse [188]. This will help reduce the effort required to create predictive models for other modeling projects.

### Significance Thresholds

In both the *Evaluating Model Performance and Power Analysis* and *Quantitative and Qualitative Analyses* sections, we use the widely adopted significance level of .05 to perform power analysis. The statistics community has debated a lot about the *P* value and its dichotomization [189-191]. Setting a threshold for the *P* value is essential for power analysis and sample size estimation [189]. In addition, to the best of our knowledge, no consensus has been reached on what the best alternative is if *P* values and statistical significance are not used [189]. Following the advice given by Amrhein et al [191], after obtaining the results of this study, we will report the actual *P* values, treat them as continuous measures of evidence against the null hypotheses rather than as parts of binary decision rules, and acknowledge that multiple independent studies are needed to provide stronger support for or against our hypotheses.

### Conclusions

Our results will help make IDM for asthma and COPD more proactive, effective, and efficient, improving outcomes and saving resources. Future studies will evaluate our methods for heart diseases, diabetes, and other diseases; deploy our methods at UWM, KPSC, and IH for IDM for asthma and COPD; and test the performance against the current IDM practice.

## Abbreviations

AUC: area under the receiver operating characteristic curve
COPD: chronic obstructive pulmonary disease
*ICD-9*: International Classification of Diseases, Ninth Revision
*ICD-10*: International Classification of Diseases, Tenth Revision
IDM: integrated disease management
IH: Intermountain Healthcare
KPSC: Kaiser Permanente Southern California
LSTM: long short-term memory
OMOP: Observational Medical Outcomes Partnership
SSH: Secure Shell
UWM: University of Washington Medicine
